# Idiopathic splenomegaly in childhood and the spectrum of RAS-associated lymphoproliferative disease: a case report

**DOI:** 10.1186/s12887-021-02508-3

**Published:** 2021-01-21

**Authors:** Geraldine Blanchard-Rohner, Robert J. Ragotte, Anne K. Junker, Mehul Sharma, Kate L. Del Bel, Henry Y. Lu, Stephanie Erdle, Alanna Chomyn, Harinder Gill, Lori B. Tucker, Richard A. Schreiber, Jacob Rozmus, Catherine M. Biggs, Kyla J. Hildebrand, John Wu, Sylvia Stockler-Ipsiroglu, Stuart E. Turvey

**Affiliations:** 1 Department of Pediatrics, British Columbia Children’s Hospital, The University of British Columbia, 950 West 28th Avenue, V5Z 4H4 Vancouver, BC Canada; 2 Children’s Hospital of Geneva, University Hospitals Geneva, Geneva, Switzerland; 3 Jenner Institute, Nuffield Department of Medicine, University of Oxford, Oxford, UK; 4 Department of Medical Genetics, The University of British Columbia, Vancouver, BC Canada

**Keywords:** RAS-associated lymphoproliferative disease, KRAS, Splenomegaly, Case report

## Abstract

**Background:**

*KRAS* (KRAS proto-oncogene, GTPase; OMIM: 190,070) encodes one of three small guanosine triphosphatase proteins belonging to the RAS family. This group of proteins is responsible for cell proliferation, differentiation and inhibition of apoptosis. Gain-of-function variants in *KRAS* are commonly found in human cancers. Non-malignant somatic *KRAS* variants underlie a subset of RAS-associated autoimmune leukoproliferative disorders (RALD). RALD is characterized by splenomegaly, persistent monocytosis, hypergammaglobulinemia and cytopenia, but can also include autoimmune features and lymphadenopathy. In this report, we describe a non-malignant somatic variant in *KRAS* with prominent clinical features of massive splenomegaly, thrombocytopenia and lymphopenia.

**Case presentation:**

A now-11-year-old girl presented in early childhood with easy bruising and bleeding, but had an otherwise unremarkable medical history. After consulting for the first time at 5 years of age, she was discovered to have massive splenomegaly. Clinical follow-up revealed thrombocytopenia, lymphopenia and increased polyclonal immunoglobulins and C-reactive protein. The patient had an unremarkable bone marrow biopsy, flow cytometry showed no indication of expanded double negative T-cells, while malignancy and storage disorders were also excluded. When the patient was 8 years old, whole exome sequencing performed on DNA derived from whole blood revealed a heterozygous gain-of-function variant in *KRAS* (NM_004985.5:c.37G > T; (p.G13C)). The variant was absent from DNA derived from a buccal swab and was thus determined to be somatic.

**Conclusions:**

This case of idiopathic splenomegaly in childhood due to a somatic variant in *KRAS* expands our understanding of the clinical spectrum of RAS-associated autoimmune leukoproliferative disorder and emphasizes the value of securing a molecular diagnosis in children with unusual early-onset presentations with a suspected monogenic origin.

## Background

The RAS family consists of three proteins all sharing a highly conserved N-terminus region: Harvey RAS (HRAS), Kirsten RAS (KRAS) and neuroblastoma RAS (NRAS). These guanosine triphosphatases bind GTP causing the activation of mitogen activated protein kinase (MAPK), phosphoinositide-3-kinase (PI3K) and Ras-like (RAL) pathways [1]. Activation leads to cell proliferation, differentiation and inhibition of apoptosis. Downstream signalling is controlled by both RAS-intrinsic GTP hydrolysis, guanine nucleotide exchange factors (GEFs) that catalyse hydrolysis and GTPase-activating proteins (GAPs) [2].

Gain-of-function variants in the three RAS genes have been found in 27 % of human cancers [3]. The disease mechanism underlying these variants is dependent on the specific defect, as some reduce intrinsic GTP hydrolysis, while others result in an insensitivity to GAP-mediated GTP hydrolysis [2]. The majority of these variants are localised to the conserved N-terminus in codons 12, 13 and 61, resulting in greater enhancement of downstream signalling, with variants in codons 12 and 13 most frequently observed for *KRAS* [4, 5]. The degree of activation and the underlying biochemical mechanism differs between variants in the three RAS genes, with the *KRAS* isoform being the most commonly disrupted in human carcinomas [6].

In 2011, somatic gain-of-function *KRAS* variants were identified as the genetic etiology of a monogenic autoimmune disorder now known as RAS-associated autoimmune leukoproliferative disorder (RALD) [7]. RALD is characterized by splenomegaly, persistent monocytosis, hypergammaglobulinemia and cytopenia, but can also include autoimmune features and lymphadenopathy [8, 9]. Here we report the discovery of a non-malignant somatic *KRAS* variant using whole exome sequencing in a minimally symptomatic then-8-year-old girl with unexplained massive splenomegaly.

## Case presentation

**Fig. 1:**
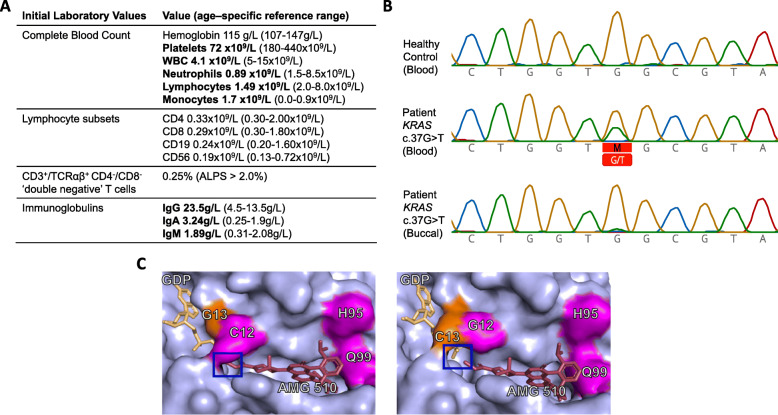
**a** Key clinical laboratory findings with age-specific reference intervals. Abnormal values are in bold. **b** Sanger sequencing of *KRAS* from DNA extracted from healthy control, patient blood, and a patient buccal swab. This identified a somatic variant in *KRAS* (NM_004985.5:c.37G > T(p.G13C)) originally discovered by whole exome sequencing on DNA derived from blood. **c** AMG 510 is a selective KRAS p.G12C inhibitor. AMG-510 binding to p.G13C KRAS was modelled using the crystallographic data of covalently-bound AMG-510 and p.G12C KRAS from PDB 6OIM [10]. The p.G13C substitution were introduced using Coot [11]. Cysteine substitution in position 12 instead of 13 of KRAS would likely affect covalent bond formation in the P2 pocket

The patient is one of twelve siblings of unrelated parents without any contributive familial history. In early childhood, she presented with easy bruising and bleeding from gums upon brushing teeth. Otherwise, she was asymptomatic and her history of infections was unremarkable. The family consulted a haematologist when she was approximately five years-old. A complete blood count revealed thrombocytopenia and lymphopenia (Fig. 1a**)** with no abnormal cells. Physical examination was notable for a firm and regular spleen with the tip palpable 7 cm below the costal margin. An abdominal ultrasound confirmed the splenomegaly with a uniform echotexture.

Inborn errors of metabolism associated with splenomegaly (e.g. Gaucher, Niemann Pick A/B and C, Tangier disease) were excluded via demonstration of normal enzyme activities, the absence of biomarkers for the respective conditions, and targeted gene sequencing. A normal bone marrow biopsy ruled out haematological malignancy. Serology showed elevated serum immunoglobulin G, A and M, C-reactive protein, tissue transglutaminase antibodies and smooth muscle antibodies, with normal levels of C3 and C4 proteins. She had generated detectable antibodies to vaccine antigens, including diphtheria and tetanus toxoids. Flow cytometry revealed low-normal numbers of CD4^+^, CD8^+^ and CD19^+^ cells. CD3^+^/TCRαβ^+^ CD4^-^/CD8^-^ ‘double negative’ (DN) T-cells were 0.25 %, making a diagnosis of autoimmune lymphoproliferative syndrome (ALPS) less likely. Key laboratory findings are documented in Fig. 1a.

Due to her atypical constellation of symptoms without a unifying explanation, singleton whole exome sequencing on DNA derived from blood was performed when the patient was 8 years old. This sequencing revealed a heterozygous missense variant in *KRAS* - NM_004985.5:c.37G > T(p.G13C), that was present in 20/47 (= 0.42) sequence reads. This is a known pathogenic variant reported in Clinvar (Accession ID: VCV000045123.2). Importantly, this specific variant had been previously linked to RAS-associated lymphoproliferative disease (RALD) as a non-malignant somatic variation [7]. Follow-up studies showed that the variant was not present in DNA extracted from a buccal swab, confirming the somatic origin of the variant (Fig. 1b**).** The patient was started on sirolimus (1 mg/m^2^/day orally based on body surface area) at age 11 years due to her progressive massive splenomegaly, thrombocytopenia and lymphopenia. Sirolimus was well-tolerated and resulted in objective improvements of the patient’s condition, including a reduction in spleen size and improved hematological parameters, and subjective improvements including increased energy and decreased abdominal circumference, reported as clothes fitting more easily. Regular follow-up has been instituted to include physical examinations, laboratory testing and imaging as guided by signs and symptoms.

## Discussion and conclusions

The variant described here (KRAS p.G13C) has been previously identified in patients who presented with an ALPS-like phenotype, but who lacked the defining increase in the CD3^+^/TCRαβ^+^ CD4^−^/CD8^−^ DN T-cell compartment, elevated biomarkers (i.e. vitamin B12), and the germline or somatic variants in *FAS*, *FASL* or *CASP10* [8, 12]. We have previously reported the same variant of somatic origin, p.G13C, in a severely affected boy who presented with Rosai-Dorfman syndrome and systemic lupus erythematosus [9].

Massive splenomegaly in children can be indicative of a severe underlying condition including infection, hemoglobinopathies, hematological malignancy, congenital anemia, inborn errors of metabolism (IEM), lysosomal storage diseases, hepatic diseases, autoimmune conditions, and lymphoproliferative disorders such as hemophagocytic lymphohistiocytosis (HLH) or autoimmune lymphoproliferative syndrome (ALPS). RALD is considered to be ALPS-related, but a distinct clinical entity that causes the dysregulation of leukocyte homeostasis [8, 9]. RALD and juvenile myelomonocytic leukemia (JMML) likely exist on a shared clinical continuum where JMML undergoes additional genetic changes responsible for malignant transformation [8, 13]. In RALD, immune dysregulation may be caused by resistance to IL2 depletion-dependent apoptosis [12]. T-cells from RALD patients exhibit a resistance to the proapoptotic BIM protein, which mediates activated-cell autonomous death (ACAD), while JMML patients have normal ACAD but defective Fas/Fas ligand-mediated activation-induced cell death (AICD) [12]. Thus, RALD-patients should be followed closely for possible transformation following accumulation of new dysplastic molecular or clonal karyotypic alternations [8].

Hematopoietic stem cell transplantation is not recommended for RALD [8]. Like other ALPS-related syndromes, potential targeted treatments include MAPK inhibitors (e.g. trametinib) and mammalian target of rapamycin (mTOR) inhibitors (e.g. sirolimus and everolimus). Efforts are ongoing to develop inhibitors against specific mutated forms of KRAS for use in cancer therapy, most notably AMG-510, which is a compound that targets the NM_004985.5:c.34G > T(p.G12C) variant in *KRAS* [10]. This drug effectively blocked downstream signalling, and reduced tumour size in a small trial of patients with lung cancer [10]. If approved, this drug may prove effective at treating RALD patients harbouring the KRAS p.G12C variant. Unfortunately, due to the high specificity of AMG-510 for KRAS p.G12C, it is unlikely to be beneficial for the patient described here, despite the close proximity of the two variants (Fig. 1c). However, like the p.G12C variant, the introduction of a nucleophilic cysteine in the p.G13C variant in our patient may also enable small molecule targeting of this mutant form of KRAS.

Developing compounds against other common KRAS variants in cancer remains of interest, most notably against p.G12D and p.G13C [5]. Due to the small number of affected individuals with rare diseases such as RALD, patients are often dependent on drugs originally developed for more common diseases. By identifying the specific molecular defect underlying RALD, we can rationally repurpose variant-specific KRAS inhibitors currently in development. This would allow us to switch from treating symptoms to targeting the underlying biochemical dysfunction, a central promise of ‘precision medicine’.

In conclusion, this case emphasizes the heterogeneous phenotype of RALD and highlights the value of molecular diagnosis for children with unusual early-onset clinical presentations that could have a monogenic origin.

## Data Availability

All relevant data are included in this manuscript and associated figures. However, if more information is required, the datasets analysed for the current study available from the corresponding author on reasonable request.

## Abbreviations

IEM: inborn errors of metabolism
HLH: hemophagocytic lymphohistiocytosis
ALPS: autoimmune lymphoproliferative syndrome
RALD: RAS-associated autoimmune leukoproliferative disorder
JMML: juvenile myelomonocytic leukemia
ACAD: activated-cell autonomous death
AICD: activation-induced cell death
mTOR: mammalian target of rapamycin
